# Vulvar Aggressive Angiomyxoma in a Postmenopausal Woman: Diagnostic Pitfalls and Surgical Implications—A Case Report

**DOI:** 10.1155/crog/5616384

**Published:** 2026-05-06

**Authors:** Melkamu Siferih, Bekalu Molla, Yemisrach Alemneh, Shiferaw Desta

**Affiliations:** ^1^ Department of Obstetrics and Gynecology, School of Medicine, Debre Markos University, Debre Markos, Ethiopia, dmu.edu.et; ^2^ Department of Pathology, School of Medicine, Debre Markos University, Debre Markos, Ethiopia, dmu.edu.et; ^3^ Department of Obstetrics and Gynecology, School of Medicine, University of Gondar, Gondar, Ethiopia, uog.edu.et; ^4^ Department of General Surgery, Debre Markos Comprehensive Specialized Hospital, Debre Markos, Ethiopia

**Keywords:** aggressive angiomyxoma, case report, Ethiopia, postmenopausal, vulva

## Abstract

**Background:**

Vulvar aggressive angiomyxoma (VAAM) is a rare, benign mesenchymal tumor with a deceptively indolent histology but locally infiltrative growth and a high risk of recurrence, typically within 3 years and occasionally more than a decade after excision. Its occurrence in postmenopausal women is exceptionally rare, often leading to delayed diagnosis and posing unique surgical challenges.

**Case Presentation:**

We report the case of a 45‐year‐old postmenopausal Ethiopian woman presenting with an 8 × 6‐cm vulvar mass initially misdiagnosed as a lipoma based on clinical findings. Absence of preoperative imaging or tissue sampling contributed to underestimation of tumor extent. Intraoperatively, the lesion demonstrated nonencapsulated infiltrative extension into deep paravaginal tissues, prompting intraoperative multidisciplinary consultation and conversion to exploratory laparotomy to exclude retroperitoneal or intrabdominal involvement. Complete wide‐margin excision was not feasible due to deep infiltration and proximity to critical neurovascular structures; therefore, maximal resection was performed. Histopathological examination confirmed aggressive angiomyxoma. At 12 months′ follow‐up, no clinical recurrence was detected; however, long‐term surveillance is ongoing.

**Conclusion:**

VAAM is a rare yet clinically demanding tumor that poses significant diagnostic and surgical challenges, particularly in postmenopausal women and resource‐limited settings. This case establishes the central role of preoperative imaging in precise tumor delineation, confirms the definitive diagnostic value of histopathological evaluation, affirms the importance of intraoperative adaptability, and supports the integration of structured, long‐term surveillance to optimize early detection of recurrence.

## 1. Introduction

Vulvar aggressive angiomyxoma (VAAM), described by Steeper and Rosai in 1983, is a rare mesenchymal tumor primarily involving the pelvis and perineum of women in their reproductive age, with peak incidence in the third decade [1]. Although histologically benign, VAAM is characterized by slow and deeply infiltrative growth and a significant propensity for local recurrence, with reported rates ranging from 30% to 72% [2]. Recurrence of VAAM most frequently occurs within the first 3 years following resection, typically emerging between 9 and 84 months posttreatment, although delayed recurrences have been documented up to 14 years later [3].

More than 80% of cases are initially misdiagnosed, frequently mistaken as lipomas, Bartholin gland cysts, or other benign vulvar masses, largely due to overlapping clinical features [4–6]. Definitive diagnosis therefore relies on histopathological examination. Characteristic microscopic features include fibroblasts embedded within a myxoid stroma, variable vascular proliferation, sparse mitotic activity, and the absence of a true capsule [4, 7].

Magnetic resonance imaging (MRI) is widely regarded as the imaging modality of choice, as it demonstrates the characteristic laminated or “swirled” internal structure on T2‐weighted sequences and allows accurate delineation of deep pelvic extension into the paravaginal space, ischiorectal fossa, retroperitoneum, or presacral region [8]. However, in low‐resource settings such as Ethiopia, limited access to MRI, fine‐needle aspiration cytology, or preoperative biopsy may result in reliance on clinical evaluation alone, increasing the risk of intraoperative diagnostic surprises and incomplete surgical planning [9–12].

This report describes an unusual presentation of VAAM in a postmenopausal Ethiopian woman, a demographic in which the tumor is exceptionally rare. Beyond documenting the case, we critically examine the diagnostic challenges, surgical decision‐making complexities, and differential diagnostic considerations in a low‐resource setting. By highlighting practical strategies for managing large, infiltrative vulvar masses without advanced imaging or readily available adjuvant therapies, this case provides insights applicable to both resource‐limited and high‐resource clinical environments.

## 2. Case Presentation

A 45‐year‐old para‐3 woman presented in December 2024 with a 3‐year history of progressively enlarging left‐sided vulvar swelling. The mass was painless, not associated with vaginal bleeding, discharge, urinary symptoms, or systemic complaints. She had been amenorrheic for 2 years. Her past medical, gynecologic, and surgical histories were unremarkable. Notably, she had no history of diabetes mellitus or other significant medical conditions.

Physical examination revealed a well‐demarcated, nontender, 8 × 6‐cm soft mass located at the junction of the left labia majora and minora, near the 4 o′clock position. The lesion was mobile, smooth, and rubbery in consistency, with clearly defined borders and no overlying skin changes, clinically consistent with a lipoma. Vital signs were stable, and systemic examination, including lymphatic, cardiovascular, respiratory, abdominal, and genitourinary assessments, was unremarkable.

Routine laboratory investigations were unremarkable. In retrospect, additional preoperative evaluation such as pelvic ultrasound, MRI, fine‐needle aspiration cytology, or incisional biopsy might have facilitated more accurate surgical planning. However, given the benign clinical appearance and limited imaging availability, such investigations were not performed prior to surgery.

**Figure 1:**
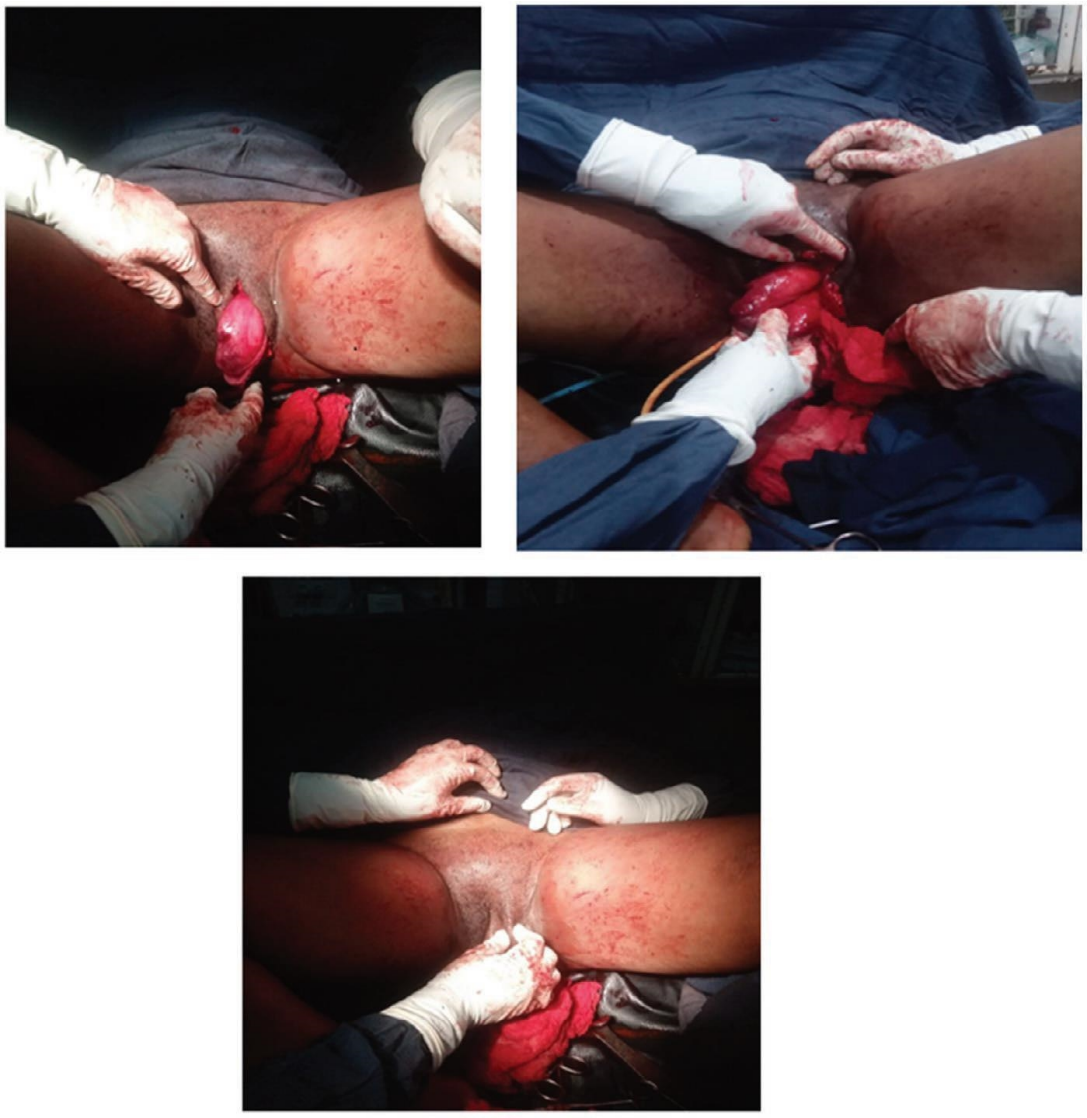
Photographs of vulvar aggressive angiomyxoma intraoperatively and postoperatively.

Under spinal anesthesia, a vertical incision was made over the most prominent aspect of the mass. Contrary to the preoperative impression of a well‐encapsulated lipoma, the tumor lacked a definable capsule and exhibited gelatinous, myxoid infiltration into surrounding paravaginal tissues. Surgical planes were indistinct, and the superior extent of the lesion could not be clearly delineated. Given concern for retroperitoneal or intra‐abdominal extension, intraoperative consultation with the general surgery team was obtained. To exclude possible intraperitoneal involvement and ensure patient safety, the procedure was converted to exploratory laparotomy under general anesthesia. No intra‐abdominal extension was identified. Upon returning to the perineal field, the infiltrative nature of the tumor into adjacent soft tissues became evident. Attempting radical wide‐margin excision would have necessitated extensive dissection with substantial risk of injury to adjacent neurovascular structures and increased morbidity. Therefore, in alignment with emerging evidence suggesting that recurrence is not strictly correlated with margin status, the surgical strategy was modified to maximal safe excision rather than radical excision (Figure 1).

Estimated blood intraoperative blood loss was approximately 350 mL, reflecting the vascularity characteristics of this tumor but remained hemodynamically manageable. The postoperative course was uneventful.

**Figure 2:**
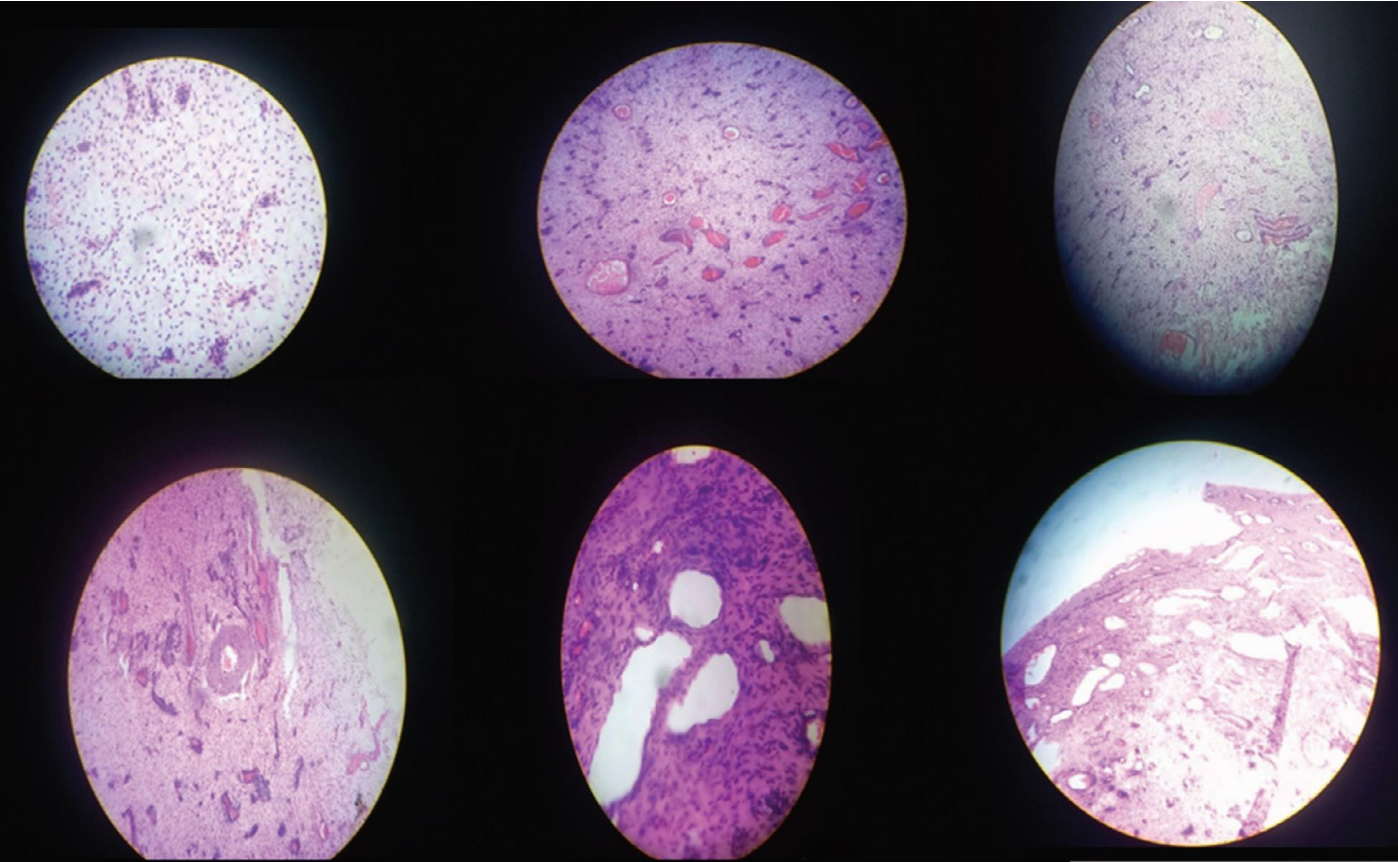
Histopathological features of vulvar aggressive angiomyxoma.

Gross examination revealed a 7 × 6 × 3‐cm soft, gray–white, myxoid mass. Microscopic evaluation demonstrated hypocellular, fibromyxoid stroma composed of spindle‐shaped cells, with small nuclei, numerous thin‐ and thick‐walled blood vessels, and entrapped nerve bundles. Mitotic figures were rare, and necrosis was absent. These findings were diagnostic of aggressive angiomyxoma (Figure 2).

The patient was counseled extensively regarding the risk of recurrence. Given that the highest recurrence rates were reported in the first 3 years, she was informed that absence of recurrence at 12 months does not exclude future relapse. A structured long‐term follow‐up plan extending beyond 3 years has been instituted. At 12 months postoperatively, there was no clinical or sonographic evidence of recurrence.

## 3. Discussion

VAAM remains a rare but clinically significant entity due to its infiltrative behavior and high recurrence rates despite benign histology. The present case is noteworthy because it occurred in a postmenopausal woman, a demographic in which VAAM is distinctly uncommon. While the majority of reported cases involve women of reproductive age, isolated cases in postmenopausal patients have been documented, suggesting that hormonal dependence may not fully explain tumor pathogenesis [10, 13]. Age‐related hormonal decline and vulvar tissue atrophy may alter tumor presentation, potentially obscuring recognition and complicating differentiation from more common benign lesions in older women. Moreover, the broad differential diagnosis of vulvar masses in this age group—including neoplastic, infectious, and dermatologic conditions—may further delay the identification of rare mesenchymal tumors [14, 15]. This observation challenges the traditional perception of VAAM as a predominantly hormonally driven tumor confined to reproductive years and accentuates the need for sustained clinical vigilance across age groups. The occurrence of VAAM in a postmenopausal woman in our setting further broadens the demographic spectrum and reinforces that chronological age alone should not reduce diagnostic suspicion.

The initial clinical diagnosis of lipoma in this case reflects the well‐documented diagnostic challenge associated with VAAM. The lesion was soft, mobile, and painless, lacking overt malignant features [4–6, 16, 17]. However, compared with typical lipomas, the prolonged progressive enlargement over 3 years and the eventual intraoperative finding of deep infiltration underscore that a lesion larger than 5 cm with slow, persistent growth should prompt further evaluation [12, 18]. Unlike lipomas, which are typically well‐encapsulated and easily dissectible, VAAM lacks a true capsule and extends along fascial planes, a distinction that only became apparent intraoperatively in this patient [10, 19]. In retrospect, preoperative imaging or biopsy might have altered surgical planning. This observation aligns with literature emphasizing MRI as the gold standard for preoperative assessment because of its characteristic “swirled” pattern and its ability to define pelvic involvement and relationships with adjacent structures such as bladder [20, 21]. In high‐resource settings, MRI not only refines diagnosis but also minimizes intraoperative uncertainty and reduces the likelihood of unexpected tumor extension. In contrast, the absence of such imaging in our context directly contributed to operative unpredictability [22]. Thus, this case exemplifies how disparities in diagnostic resources can materially influence surgical strategies.

The decision to convert to exploratory laparotomy was driven by intraoperative uncertainty regarding the superior extent of the tumor. While such conversion is rarely described in high‐resource settings where preoperative MRI is routinely available, this case illustrates how the absence of advanced imaging may necessitate intraoperative adaptability [23, 24]. The inability to delineate safe dissection planes and concerns regarding possible retroperitoneal extension required a safety‐first approach, prioritizing exclusion of intraperitoneal involvement over procedural expediency. Importantly, no intraperitoneal extension was identified, highlighting that deep pelvic infiltration does not necessarily equate to abdominal involvement [25, 26]. This distinction is clinically relevant, as the overestimation of tumor spread may lead to unnecessary radical procedures, whereas underestimation risks incomplete resection or structural injury. This experience reinforces the critical role of multidisciplinary collaboration in managing complex pelvic tumors, particularly in settings with limited preoperative staging resources [27, 28].

From a surgical standpoint, complete wide‐margin excision has historically been advocated [28, 29]. However, accumulating evidence indicates that recurrence may occur even after negative margins and, conversely, that patients with positive margins do not invariably experience relapse [30]. This observation challenges the traditional oncologic paradigm of radical resection. Several contemporary series suggest that microscopic margin status does not consistently predict recurrence, implying that tumor biology and hormonal responsiveness may play more significant roles than previously appreciated [28, 31]. In our case, maximal safe excision was favored over aggressive radical surgery to preserve function and minimize morbidity. This decision was guided by the recognition that radical resection in the vulvoperineal region may result in substantial functional and cosmetic sequelae without clear evidence of recurrence reduction. Such an approach is increasingly supported in contemporary literature and may be particularly relevant in limited‐resource clinical contexts where reconstructive options and perioperative support are constrained [29, 31].

Recurrence rates reported in the literature range from 30% to 72%, with most recurrences occurring within 3 years, although delayed recurrence up to 14 years has been described [31, 32]. Consequently, labeling a patient recurrence‐free at 12 months would be premature. The current follow‐up period in our case represents an early postoperative phase rather than definitive oncologic control. Long‐term surveillance extending beyond 3–5 years is strongly recommended. In settings where serial MRI is not readily available, structured clinical follow‐up supplemented by ultrasound may represent a pragmatic alternative for monitoring recurrence [31, 33, 34].

Hormonal receptor positivity, particularly estrogen and progesterone receptors, has been reported in many cases of VAAM, supporting the potential role of hormonal therapy in disease management. Medical treatments such as gonadotropin‐releasing hormone (GnRH) analogues and, more recently, aromatase inhibitors have been used in selected patients with recurrent, residual, or unresectable tumors, with several reports describing tumor regression or stabilization [35–37]. Aromatase inhibitors may be particularly relevant in postmenopausal women because they suppress peripheral estrogen production [38–40]. In the present case, adjuvant hormonal therapy was not administered due to the limited availability of these medications in our setting. Nevertheless, the potential benefit of hormonal therapy—particularly aromatase inhibitors in postmenopausal patients—should be considered in cases of recurrence or unresectable disease.

Histopathology ultimately confirmed the diagnosis of VAAM in this patient. The tumor demonstrated the characteristic hypocellular myxoid stroma with numerous thin‐ and thick‐walled vessels and spindled‐shaped stromal cells, findings consistent with previously described histomorphological criteria [10, 41–47]. Accurate pathological evaluation remains essential, particularly in clinical environments where imaging resources are limited and clinical presentation may be misleading.

This case contributes incremental yet meaningful insight by illustrating how diagnostic delay, absence of preoperative imaging, and intraoperative unpredictability can influence surgical decision‐making, particularly in postmenopausal women, who may present with atypical or rapidly enlarging vulvar masses. It highlights the importance of maintaining a broad differential diagnosis for large vulvar masses and supports a pragmatic, function‐preserving surgical strategy guided by contemporary evidence. These observations may offer practical lessons for clinicians managing similar tumors in resource‐constrained settings.

This report represents one of the few documented cases of VAAM in Ethiopia [22]. The case highlights broader structural challenges in managing rare tumors. Limited imaging diagnostic capacity, constrained access to specialized surgical expertise, and restricted availability of adjuvant therapies highlight the need for targeted capacity building in diagnostic and oncologic infrastructure. Strengthening referral networks and long‐term surveillance systems may be particularly important for improving outcomes in similar settings.

The case report has several limitations. First, it describes a single patient, limiting generalizability to other populations and settings. Second, advanced preoperative imaging such as MRI or CT scan was not available, restricting precise delineation of tumor extent prior to surgery. Third, adjuvant hormonal therapy could not be administered due to lack of availability, preventing evaluation of potential benefits in reducing recurrence risk. Finally, follow‐up was limited to 12 months, which may be insufficient to capture late recurrences commonly reported in VAAM. Despite these limitations, this report contributes valuable insights into the presentation, diagnosis, and management of VAAM in postmenopausal women in low‐resource settings.

## 4. Conclusion

VAAM, although rare, should remain in the differential diagnosis of large, slowly enlarging vulvar masses, even in postmenopausal women. Preoperative imaging and biopsy should be considered for lesions exceeding 5 cm or demonstrating sustained growth. In settings when imaging is unavailable, surgeons must anticipate possible deep infiltration and remain adaptable intraoperatively. Maximal safe excision rather than radical resection may present an appropriate balance between oncologic control and functional preservation. Structured long‐term follow‐up is essential before excluding recurrence.

**Nomenclature**
CT: computed tomography
GnRH: gonadotropin releasing hormone
HBsAg: hepatitis B surface antigen
MRI: magnetic resonance imaging
VAAM: vulvar aggressive angiomyxoma

## Author Contributions

M.S. conceived the case report. M.S. and S.D. were involved in the patient care. All authors made substantial contributions to the study design, data collection, literature review, analysis, and interpretation of this case report. They were actively involved in drafting, revising, and critically reviewing the manuscript.

## Funding

No funding was received for this manuscript.

## Disclosure

All authors approved the final version for publication, agreed on the chosen journal for submission, and take full responsibility for the accuracy and integrity of the work.

## Consent

In accordance with institutional guidelines, ethical approval was not required for this case report. Written informed consent was obtained from the patient for participation and for the publication of the clinical details, anonymized data, and accompanying images. The patient was informed of the purpose and nature of the report, potential implications of publication, and their right to withdraw consent at any time without impact on their care. The signed consent is documented in the patient′s medical record, and a copy is available for review by the journal′s editor‐in‐chief upon request.

## Conflicts of Interest

The authors declare no conflicts of interest.

## Data Availability

All relevant data underlying the findings of this case report are included in the article.
